# Early Childhood Caries-Related Knowledge, Attitude, and Practice: Discordance between Pediatricians and Dentists toward Medical Office-Based Prevention in Taiwan

**DOI:** 10.3390/ijerph15061067

**Published:** 2018-05-24

**Authors:** Shinechimeg Dima, Wei-Jen Chang, Jung-Wei Chen, Nai-Chia Teng

**Affiliations:** 1 School of Dentistry, College of Oral Medicine, Taipei Medical University, 250 Wu-Hsing Street, Taipei 110, Taiwan; d204103003@tmu.edu.tw (S.D.); cweijen1@tmu.edu.tw (W.-J.C.); 2 Department of Dentistry, Shuang Ho Hospital, New Taipei 235, Taiwan; 3 School of Dentistry, Department of Pediatric Dentistry, Loma Linda University, Loma Linda, CA 92350, USA; jwchen@llu.edu; 4 Department of Dentistry, Taipei Medical University Hospital, 250 Wu-Hsing Street, Taipei 110, Taiwan

**Keywords:** early childhood caries, knowledge, attitude and practice, medical office-based prevention

## Abstract

The aim of this study was to assess the knowledge, attitude, and practice regarding early childhood caries (ECC) prevention and implementation of medical setting-based caries prevention among pediatricians and dentists in Taiwan. Data were collected from currently practicing pediatricians and general and pediatric dentists using self-administered questionnaires. A total of 301 questionnaires were completed by the pediatricians (*n* = 105), general dentists (*n* = 117), and pediatric dentists (*n* = 79). The pediatric dentists obtained significantly higher knowledge and practice scores than the general dentists and pediatricians (*p* < 0.0001). The pediatricians’ attitude score related to engaging physicians in medical office-based caries prevention was significantly higher than the attitude scores of the general and pediatric dentists (*p* < 0.05). A Spearman rank correlation analysis indicated a significant positive correlation between knowledge and practice among the general dentists (r_s_ = 0.271, *p* < 0.01) and pediatricians (r_s_ = 0.262, *p* < 0.01). The correlation between knowledge and attitude among the pediatricians was significantly positive (r_s_ = 0.242, *p* < 0.05). Attitude and practice among the pediatricians were significantly positively correlated (r_s_ = 0.271, *p* < 0.01). Pediatricians lacked ECC-related knowledge; however, they had a more positive attitude toward medical office-based prevention when they had a higher level of knowledge. Oral health-related education for pediatricians is necessary if such medical office-based caries prevention programs are to be implemented in Taiwan.

## 1. Introduction

Early childhood caries (ECC) is defined as the “presence of one or more decayed (noncavitated or cavitated lesions), missing (due to caries), or filled tooth surfaces in any primary tooth in a child aged 71 months or younger” [1]. ECC has significant adverse consequences on a child’s health and overall well-being, including increased risk of future caries, hospitalizations, emergency room visits, and missed school days.

ECC is a major public health concern in Taiwan. The prevalence of caries increased with age among Taiwanese preschool children, with a prevalence of 7.08%, 31.4%, 51.55%, 78.05%, and 79.32% among children aged 1–2, 2–3, 3–4, 4–5, and 5–6 years, respectively, in 2011 [2]. Tsai et al. reported high numbers of untreated caries among children in Taiwan with a mean deft of 0.14 at age 2, 2.58 at age 3, 4.41 at age 4, 6.94 at age 5, and 7.31 at age 6 [3]. By age 6, 89% of children had caries.

Fluoride varnish has been widely accepted by dental experts as a crucial component of preventive dental health care in preschool children [4,5]. Fluoride varnish applied twice a year, along with counseling of the caregiver regarding diet and tooth brushing, has proved to be efficacious in reducing the incidence of dental caries in children [6]. In 2004, the Taiwan government launched a preventive fluoride varnish program aimed at reducing the disease burden of caries among preschool children. Currently, National Health Insurance (NHI) provides fluoride varnish application to children younger than 6 years with an interval of at least 6 months. The preventive fluoride varnish treatment service is provided only by licensed dental practitioners in dental offices, which are reimbursed by the government for the service.

The preventive services by dentists comprise an oral examination of the child, caries risk assessment, oral health counseling for parents, and application of fluoride varnish. Although over 95% of dentists practicing in various settings are contracted to provide this treatment in Taiwan, since its implementation, most children have not received fluoride varnish. Statistics indicated that only 0.5% to 34% of children aged 0 to 4 were administered fluoride varnish treatment twice a year in 2012 [2]. The rate of fluoride use was relatively high among children aged 4–5 years (43.3%), whereas younger children had a lower probability of receiving fluoride varnish application [7]. Among children with disability, up to 9.84% received fluoride varnish treatment, and the use decreased with an increase in the level of disability [8]. Therefore, an increase in fluoride varnish use for preventing caries among preschool-aged children in Taiwan is necessary.

Primary care physicians and pediatricians establish early relationships with young children and their parents. They frequently examine infants and young children in the early years of life when prevention is critical and lifelong habits are being established. As previously reported, 89% of infants and children aged 1 year had medical office-based physician visits annually, compared with only 1.5% who had dental visits [9]. Family physicians and pediatricians often examine children up to six times before the age of 2 years; thus, these appointments must be considered opportunities to increase awareness of oral health evaluations, screen young children for caries risk, and refer children to dental care [10]. Preventive measures initiated during the first years of life can significantly reduce the risk of ECC and the need for expensive restorative procedures later in life. Considering that preschool-aged children are significantly more likely to visit a physician’s office than a dentist’s office and the current need to increase the use of caries prevention services, the interest among pediatricians in Taiwan in incorporating fluoride varnish application for caries prevention into their patients’ medical visits has been increasing in recent years. By acquiring skills for applying preventive strategies and appropriately referring patients to dentists, primary care physicians and pediatricians may help eliminate oral health disparities. However, no research has been conducted to assess the degree to which physicians in Taiwan are knowledgeable in aspects of oral health in children. Moreover, evaluating the current knowledge and practice regarding caries prevention of dentists in Taiwan is necessary, prior to considering the involvement of other health professionals in caries prevention activities. To date, limited literature is available on the knowledge, attitude, and practice (KAP) aspects of caries prevention among dentists in Taiwan [11]. 

The present study analyzed the ECC-related KAP of dentists and pediatricians and identified the pathways through which the knowledge and practice of medical and dental professionals in Taiwan affect their attitude toward medical office-based caries prevention in Taiwan. 

## 2. Methods

### 2.1. Study Design and Setting

This cross-sectional quantitative study was conducted in Taipei City, Taiwan. Data were collected from specialized pediatric health care physicians and general and pediatric dentists in Taiwan. Participants were required to be currently practicing in a public hospital, university hospital, or private practice setting in Taiwan. Full- and part-time practicing professionals were eligible to participate in the study. Two types of approaches were used by trained investigators to recruit participants for the study: potential participants were either visited on-site at their respective hospitals during nonclinical hours by appointment or recruited during professional annual conference meetings during the study period. All potential participants were provided with information on the purpose of the survey. Only those who provided verbal consent were enrolled in the study. All response data of the participants were analyzed anonymously. No incentives were provided to the respondents. The study participants sample represented as much like the population. Sample reflects the characteristics of the population in gender, age, years after graduation and practice type of each group.

### 2.2. Data Collection

Data were collected from the respondents using a standardized self-administered questionnaire, distributed as hard copies by trained investigators. The questionnaire was designed to collect data on the participants’ KAP regarding ECC and its prevention. The knowledge and practice parts of the questionnaire were developed using information from the literature on caries prevention in children, including the current clinical guidelines and policies from the American Academy of Pediatrics and American Academy of Pediatric Dentistry (AAPD, Chicago, IL, USA) [9,12]. The attitude questions were framed using the literature regarding barriers to the implementation of medical office-based caries prevention [13]. KAP construction guides were used to develop and conduct the survey [14]. Researchers explained the purpose of the study to the respondents and instructed them to fill in the questionnaire anonymously. The dentist and pediatrician versions of the questionnaire consisted of 52 and 54 items, respectively.

The questionnaire consisted of five parts. The first section concerned the demographic and occupational characteristics of the study participants and had 11 and 13 questions in the dentist and pediatrician versions, respectively. The sociodemographic data included gender, age in categories (24–34, 35–44, 45–54, 55–64, and ≥65 years), practice hours (part time or full time), years after graduation (<5, 5–10, 10–15, 15–20, 20–25, and >25 years), practice region (urban, suburban, or rural), and age of patients in categories (0–3, 3–6, 6–12, and >12 years). The second section of the questionnaire was designed to evaluate the participants’ knowledge related to ECC and prevention in children. This section consisted of 10 multiple-choice, close-ended questions, including the time of tooth eruption, time of a child’s first dental visit, and methods of fluoride usage in caries prevention (e.g., toothpaste, mouth rinse, tablet, and fluoride varnish). The third section was dedicated to exploring practices regarding caries prevention, including the performance of the following six practice activities: inquiring about the bottle, conducting oral examination, assessing caries risk, assessing fluoride intake, counseling on toothbrushing, and counseling to parents. The fourth section consisted of four questions on the current fluoride varnish program in Taiwan and a question regarding the application of fluoride varnish treatment to prevent caries by physicians, “Do you agree that physicians can apply fluoride varnish to prevent ECC in children?”. The fifth section comprised 13 items regarding attitude toward potential barriers that may be encountered in implementing medical office-based caries prevention programs, including physicians’ knowledge, confidence, learning, and time factors [15].

Cronbach’s Alpha was used to assess the internal consistency of the questionnaire. The Cronbach’s Alpha coefficients of knowledge, attitude and practice (KAP) domains were 0.57, 0.76 and 0.89, respectively.

## 3. Scoring

Knowledge questions with correct responses were scored 1 and incorrect answers were scored 0. We combined “do not know” responses with incorrect answers and scored them as 0. The sum of all 10 knowledge scores was used to quantitatively describe the knowledge of each participant. For each of the six caries prevention practice activities, the participants from the general and pediatric dentist groups were asked to grade the frequency with which they practice each activity on a scale of 1 to 5, with 1 representing “very rarely” and 5 representing “very often.” For pediatricians, a practice section was designed to grade their willingness to perform preventive activities if those activities are included in their routine practice on a scale of 1 to 5, with 1 representing “very unwilling” and 5 represented “very willing.” The sum of all six practice scores was used to quantitatively describe the practice of the participants. To analyze the distribution of responses to practice questions in the three groups, “often” and “very often” responses and “uncertain,” “rarely,” and “very rarely” responses of dentists were grouped together. In the pediatricians’ version, “willing” and “very willing” responses and “uncertain,” “unwilling,” and “very unwilling” responses were grouped together. Attitude was assessed on three-point scales (“agree” = 1, “uncertain” = 2, and “disagree” = 3) to statements regarding barriers in implementing medical office-based caries prevention. The sum of all 13 attitude scores was used to quantitatively describe the attitude of the participants. Higher scores denoted a more positive attitude toward engaging physicians in preventing caries in medical offices. The maximum possible scores were 10, 30, and 39 for knowledge, practice, and attitude, respectively.

## 4. Ethical Consideration

The research was conducted in accordance with the tenets of the Declaration of Helsinki and was approved by the Taipei Medical University Joint Institutional Review Board (Reference: N201608007). This study got approval to have informed consent waiver. The questionnaire was anonymous and the participants were informed about their right to withdraw from the study at any stage. All data were analyzed anonymously.

Statistical Analysis

The demographic and baseline characteristics of the study participants were summarized, and differences in study variables were explored using the chi-square test. The KAP scores of the three groups were reported as means and compared between groups by the *t* test. The responses to the question “Do you agree that physicians can apply fluoride varnish to prevent ECC in children?” were summarized and the chi-square test was used for analysis. The Spearman rank correlation was used (r_s_) to calculate the correlation values between the KAP scores of the three participant groups. Univariate logistic regression analysis was used to determine odds ratios (ORs) and 95% confidence intervals (CIs) for attitude item responses to the aforementioned question “Do you agree that physicians can apply fluoride varnish to prevent ECC in children?” The level of significance was set at 0.05. All data were analyzed using SPSS version 21.0 (IBM Inc., Armonk, NY, USA).

## 5. Results

### 5.1. Study Population and Sociodemographic Characteristics

A total of 315 questionnaires were distributed; of these, 14 were excluded because of nonresponse or incomplete data. Consequently, the data of 301 participants, namely 117 (38.8%) general dentists, 79 (26.2%) pediatric dentists, and 105 (34.8%) pediatricians, were studied. The characteristics of the study participants among the three groups of professionals are summarized in Table 1. Regarding the sociodemographic characteristics, gender (*p <* 0.0001), age (*p <* 0.0001), years after graduation (*p <* 0.01), practice type (*p* < 0.0001), and age of patients (*p* < 0.01) were significantly different among the three groups. Most pediatricians (66.7%) practiced in public hospitals, whereas a majority of both groups of dentists were engaged in private practice. In the pediatrician group, 57% of the respondents had taken some oral health courses during their medical school term (27.6%), residency training (18.1%), and continuing medical education (29.5%).

### 5.2. Caries Prevention-Related KAP Assessment

Table 2 summarizes the responses of the three groups of professionals to the KAP questions. For the fluoride-related questions, most of the pediatricians were more likely than the general and pediatric dentists to answer incorrectly. The general dentists obtained significantly fewer correct answers compared with the pediatric dentists (*p* < 0.05) on the questions regarding the first dental visit of a child; fluoride mouth rinses, supplements, and tablets; and topical fluoride. The assessment of oral health preventive practice questions revealed that only half of the general and pediatric dentists (50% and 45.6%, respectively) often or very often inquired about parents’ oral health during pediatric dental examination. Compared with the pediatric dentists (75.9%), only 34.5% of the general dentists assessed fluoride intake in their young patients. Furthermore, general dentists significantly less frequently inquired about bottle usage and caries risk assessment compared with pediatric dentists (*p* < 0.05). More than half of the pediatricians were willing or strongly willing to inquire about bottle usage, examine child’s teeth, assess the child’s caries risk, counsel the parents regarding tooth brushing, and inquire about parents’ oral health if oral health prevention activities were included in their routine practice. Only 35% of the pediatricians were willing to the assess fluoride intake in their patients. For the attitude regarding medical office-based prevention questions, 76% of the pediatricians disagreed that “physicians have no time for dentist referral” and 50% disagreed that “physicians have no time for counseling parents” and “patients are too young and uncooperative in medical offices”, respectively. Only 26.9% of the pediatricians disagreed that “fluoride varnish is difficult for physicians to apply”, in contrast to the 45.3% of the general and 35.4% of the pediatric dentists who disagreed with the item.

The mean KAP scores of the three groups of participants are summarized in Table 3. The pediatric dentists had significantly higher knowledge scores in ECC prevention than those of the general dentists and pediatricians (*p* < 0.0001). The general dentists had significantly higher knowledge scores than those of the pediatricians (*p* < 0.0001). The results of ECC prevention practice assessment indicated that the pediatric dentists obtained significantly higher scores than those of the general dentists and pediatricians (*p* < 0.0001). By contrast, the difference between the mean practice scores of the general dentists and pediatricians was not statistically significant. The attitude score of the pediatricians related to engaging physicians in caries prevention was significantly higher than those of the general and pediatric dentists (*p* < 0.05).

### 5.3. Current Fluoride Varnish Program and Potential Application by Physicians

More than 95% of the respondents among the general and pediatric dentist groups knew about the current NHI-administered fluoride varnish program and correctly identified its application time as twice a year (data not shown).

Among the general dentists, 65.8% disagreed that physicians can apply fluoride varnish in children to prevent ECC (Table 4). Among the pediatric dentists, only 20.3% agreed that physicians could apply fluoride varnish, whereas 79.7% disagreed. By contrast, a majority of the pediatricians (58.3%) agreed that physicians can apply fluoride varnish in children. The differences in the response levels among the three groups of participants were statistically significant (*p* < 0.0001).

### 5.4. Correlations between Knowledge, Attitude, and Practice

Table 5 presents the correlations of knowledge, attitude, and practice among the three groups of participants. The Spearman rank correlation of KAP scores revealed a significant positive correlation between knowledge and practice among the general dentists (r_s_ = 0.271, *p* < 0.01) and pediatricians (r_s_ = 0.262, *p* < 0.01). A negative correlation was found between knowledge and attitude among the general dentists; however, the correlation was not statistically significant (r_s_ = −0.178, *p* = 0.056). The correlation between knowledge and attitude among the pediatricians was significantly positive (r_s_ = 0.242, *p* < 0.05). Attitude and practice were significantly positively correlated in the pediatricians (r_s_ = 0.271, *p* < 0.01). 

### 5.5. Potential Barriers in Implementing Medical Office-Based Prevention by Physicians

Table 6 summarizes the results of a univariate binary logistic regression analysis on the association of attitude items and the response to the statement “Do you agree that physicians can apply fluoride varnish?”. Among the general dentists, participants who agreed with the items stating that dentists should perform ECC prevention practice activities (OR = 4.43; 95% CI: 1.32–14.76), learning those activities is difficult for physicians (OR = 7.5; 95% CI: 2.3–23.44), learning those activities is time consuming for physicians (OR = 4.02; 95% CI: 1.55–10.45), fluoride varnish is difficult for physicians to apply (OR = 6.85; 95% CI: 2.31–20.26), physicians have no time for oral screening (OR = 2.96; 95% CI: 1.21–7.26), physicians have no time for caries risk assessment (OR = 5.12; 95% CI: 2.07–12.68), physicians have no time for fluoride varnish application (OR = 5.38; 95% CI: 2.01–14.38), physicians have no time for dentist referral (OR = 3.75; 95% CI: 1.43–9.83), physicians are not sufficiently knowledgeable (OR = 5.76; 95% CI: 2.17–15.27), physicians are not sufficiently confident (OR = 3.69; 95% CI: 1.35–10.07), and patients are too young and uncooperative in medical offices (OR = 6.76; 95% CI: 2.39–19.1) were significantly more likely to disagree that physicians can apply fluoride varnish to prevent ECC in children (*p* < 0.05) than were those who disagreed to the respective attitude items. Moreover, the general dentists who were uncertain regarding whether physicians have time for dentist referral (OR = 7.51; 95% CI: 1.59–35.39) were significantly more likely to disagree that physicians can apply fluoride varnish (*p* < 0.05) compared with those who disagreed with the item. Among the pediatric dentists, those who agreed that preventive activities are not sufficiently important to include in physicians’ practice (OR = 0.06; 95% CI: 0.006–0.64), fluoride varnish is difficult for physicians to apply (OR = 16.1; 95% CI: 1.9–137.1), physicians have no time for caries risk assessment (OR = 6.09; 95% CI: 1.76–21.04), physicians have no time for fluoride varnish application (OR = 5.4; 95% CI: 1.26–23.04), and physicians have no time for counseling parents (OR = 3.6; 95% CI: 1.02–12.69) were significantly more likely to disagree that physicians can apply fluoride varnish to prevent ECC in children (*p* < 0.05) than were those who disagreed with the attitude items. The pediatricians who agreed that preventive activities are not sufficiently important to include in pediatric visits (OR = 12.7; 95% CI: 1.48–108.76), physicians have no time for fluoride varnish application (OR = 16.0; 95% CI: 1.98–129.29), and physicians are not sufficiently confident (OR = 8.5; 1.77–40.71) were significantly more likely to disagree that physicians can apply fluoride varnish (*p* < 0.05) than were those who disagreed with the attitude items. Furthermore, the pediatricians who were uncertain regarding whether physicians have time for fluoride varnish application (OR = 13.17, 95% CI: 1.54–112.05), physicians have no time for oral screening (OR = 3.08; 95% CI: 1.09–8.67) and whether physicians are sufficiently confident (OR = 7.08, 1.4–35.7) to apply varnish were also significantly more likely to disagree that physicians can apply fluoride varnish (*p* < 0.05) than were those who disagreed with these items.

## 6. Discussion

After more than a decade since the Bureau of NHI launched the fluoride varnish application program for children younger than 6 years to prevent ECC, the use of fluoride varnish application among the young population in Taiwan has been increasing each year; however, the rate remains below the desired value. The findings of the present study suggest that pediatric health professionals in medical offices have a relatively positive attitude toward and willingness to practice caries prevention activities if those activities are included in their routine practices. Interestingly, the attitude level among dental professionals regarding the engagement of physicians in medical office-based caries prevention was significantly lower than that of pediatricians. Discordance in attitudes toward physicians’ engagement in caries prevention activities was largely explained by the knowledge level of the participants.

The present study demonstrated an overall lack of oral health knowledge among the pediatricians. More than half of the pediatricians correctly responded to only two knowledge items; specifically, they correctly indicated that having meals in close succession entails a risk of caries (62.9%) and identified the timing of the child’s first dental visit (74.3%). These findings are similar to those in other investigations on pediatricians from Canada, the United States, Iran, Italy, Saudi Arabia, Brazil, and the Netherlands regarding the knowledge of physicians and primary care providers about early childhood caries and infant oral health [16,17,18,19,20]. We found that the pediatricians’ knowledge level was positively correlated with their willingness to perform caries prevention practices. However, only 35% of the pediatricians were willing to assess fluoride intake as part of caries prevention for their patients. This finding may be attributed to pediatricians’ insufficient knowledge on fluoride usage for preventing caries.

Our findings indicate that despite the generally low level of knowledge in caries prevention, pediatricians with higher knowledge levels were significantly more likely to possess positive attitudes toward medical office-based caries prevention and be willing to perform caries prevention practices. In the present study, more than half of the pediatricians (58%) agreed that they could apply fluoride varnish to prevent ECC in children. Our findings are in accordance with previous studies that reported a more positive attitude toward pediatricians’ role in preventing oral diseases in pediatricians with a higher level of knowledge. Di Giuseppe et al. described physicians more knowledgeable in oral health as being more likely to play a role in promotion of children’s oral health [18].

Oral health care and caries prevention in children should incorporate caries risk assessment, individualized preventive strategies, and anticipatory guidance [12,21]. Establishing the periodicity of supervision during care intervals and determining age-appropriate “care paths” are based on the risk of disease in each individual. An infant oral health visit consists of a six-step protocol, comprising caries risk assessment, proper positioning of the child, age-appropriate tooth brushing prophylaxis, oral clinical examination, fluoride varnish treatment, and anticipatory guidance and counseling. For optimal outcomes, caries risk should be assessed as early as possible, and preferably before the onset of the disease process. A lack of knowledge and information regarding caries prevention and preventive interventions prior to fluoride varnish application may cause pediatricians to acknowledge fluoride varnish application as a straightforward activity, which leads them to believe that they can perform it. Furthermore, for those pediatricians who disagreed that they can apply fluoride varnish, a lack of confidence and uncertainty concerning whether oral screening and fluoride varnish application take time were found to be significant factors affecting their perception of applying fluoride varnish. These uncertain responses may be related to the low level of knowledge and lack of training regarding oral health care among pediatricians. Positive associations between physicians’ confidence and their likelihood of performing oral health practices have been identified in the United States [22,23]. Lewis et al. reported that although pediatricians clearly support preventive oral health, the most cited barrier to these pediatricians’ further involvement in oral health was the lack of training [13]. In the present study, only 58% of the pediatricians had some exposure to oral health courses. Another study by Herndon and colleagues demonstrated that oral health training indirectly influences physicians’ pediatric oral health practices by increasing their confidence in activities such as advising parents and performing oral health screening and risk assessment [24]. Efforts to engage physicians in oral health training will promote physicians’ confidence and increase the likelihood of their performing preventive oral health care practices [25]. The results of our study indicated a positive association between the knowledge and attitude of pediatricians. Thus, if appropriate education and training are provided, pediatricians will support their role in caries prevention and will be willing to provide caries prevention services. Therefore, efforts are necessary to expose pediatricians in Taiwan to oral health-related education and provide them with training to increase their knowledge and confidence in practicing oral health preventive measures in children if a medical office-based caries prevention program is to be implemented in Taiwan.

The present study demonstrated a good overall knowledge level among general and pediatric dentists in Taiwan. By contrast, the score in attitude toward medical office-based caries prevention was significantly lower in the two groups of dentists than in the pediatricians. The knowledge level of the general dentists had a negative correlation with their attitude toward engaging physicians in providing caries prevention measures to children; however, this correlation was not statistically significant. A majority of the general dentists (65.8%) and pediatric dentists (79.7%) disagreed that physicians can apply fluoride varnish to prevent ECC in children. It appears that when dentists are knowledgeable in aspects of oral health in children and their challenges, they acknowledge its importance more and are likely to practice the preventive activities by themselves; however, they consider other health professionals unable to perform these activities. Dentists are aware of necessary procedures prior to fluoride varnish application, including oral examination and caries risk assessment, and necessary oral hygiene instructions that should be provided afterward to the caregivers of children [26]. Although the correlation was not statistically significant, it may explain why the general dentists who had higher knowledge levels, had a negative attitude toward whether ECC prevention can be performed by pediatricians. It is expected that the pediatric dentists possessed the highest knowledge and practice levels regarding oral health in children. Thus, the overall low level of attitude among the pediatric dentists was presumably caused by their high knowledge levels. In a previous survey among AAPD members, pediatric dentists responded that the AAPD should focus its efforts on educating pediatricians and primary care physicians regarding the value of early dental evaluations [27].

In our study, a translation of knowledge into practice was observed. A previous systemic review demonstrated that dental education and training has emerged as the most important factor affecting dentists’ attitude toward how they conduct their activities [28]. Dentists who were continuously engaged in their professional and educational development were more open to the new demands of the profession and more likely to include prevention in their daily routine. This finding is consistent with our results regarding general dentists because their caries prevention practice levels were positively correlated with their ECC-related knowledge levels. General dentists with higher knowledge levels and information on children’s oral health performed caries prevention activities frequently in their practice. However, this correlation was not observed among pediatric dentists. We found that pediatric dentists frequently practiced caries prevention measures regardless of their knowledge level.

Although the present study revealed that general and pediatric dentists in Taiwan were knowledgeable overall in aspects of oral health in children, differences in knowledge concerning certain items were observed between dental professionals; specifically, the knowledge level of general dentists regarding fluoride usage in caries prevention was lower. Only 42.7% of the general dentists responded correctly to the knowledge item related to fluoride tablets. Therefore, the knowledge level of general dentists regarding fluoride usage for ECC prevention should be improved and updated according to the current clinical guidelines to acquire consistent knowledge to further enable and encourage them to provide their patients with optimal preventive care. Our study is not the first to indicate gaps in fluoride usage-related knowledge between general and pediatric dentists. A previous study by Narendran et al. demonstrated a knowledge gap between dentists in the aspects of excessive chronic fluoride exposure, the fluoride supplement dosage, and inappropriate prescription practices; thus, they emphasized strengthening educational interventions at the undergraduate and practitioner levels [29]. 

There are a few limitations in this study. First, the study had a small sample size. We could not identify the potential confounding variables may due to the limited sample size. Second, pediatricians’ practice behaviors were self-reported according to the willingness to perform the activities; thus, they might not reflect the actual practice patterns. Third, overall KAP surveys have inherent limitations: for instance, respondents may be primed to provide certain responses, and the multiple-choice format of the questionnaire may direct them to socially desirable answers.

The preventive care policy for applying fluoride varnish in children in Taiwan still requires aggressive promotion, and additional efforts must be made to increase the application of caries prevention interventions among preschool-aged children. Partnerships with other health care professionals for providing preventive care for high-risk populations is crucial to improving oral health outcomes in the future. Caries risk assessment by a pediatrician or family physician and referral of high-risk children to dentists would reduce the disease burden under most plausible scenarios. Pediatricians and dentists must work together to improve the quality of preventive oral health care available to all young children. The findings of this study are expected to contribute to the understanding of dentists’ and physicians’ current knowledge, attitude, and practices regarding oral health in children in Taiwan. The results of this study indicate the need to improve oral health-related knowledge in pediatricians if such medical office-based caries prevention programs are to be implemented in Taiwan. This information may then be used as a useful reference for planning and decision-making for improving the oral health care provided to the younger population in Taiwan.

## 7. Conclusions

Overall, pediatricians in Taiwan lacked ECC-related knowledge; however, they had a more positive attitude toward medical office-based prevention when they had a higher level of knowledge. By contrast, although general and pediatric dentists were knowledgeable overall regarding the ECC prevention aspects, they had a negative attitude toward medical office-based prevention. Our results revealed that a majority of the pediatricians agreed that physicians could apply fluoride varnish to children’s teeth to prevent dental caries, which comprises a crucial part of the caries prevention program for preschool-aged children in Taiwan. By contrast, the dentists disagreed that physicians can perform the activity. A lack of time and confidence among pediatricians were significant factors that deterred them from providing caries prevention fluoride treatments to children. Therefore, efforts are required to expose pediatricians in Taiwan to oral health-related education and provide them with training to increase their knowledge and confidence in practicing preventive measures for oral health in children if medical office-based caries prevention programs are to be implemented in Taiwan.

## Figures and Tables

**Table 1:** General characteristics of the participants.

		General Dentists	Pediatric Dentists	Pediatricians	*p* Value
		*n* (%)	*n* (%)	*n* (%)	
Gender					<0.0001
Male		70 (59.8)	23 (29.1)	66 (62.9)	
Female		47 (40.2)	56 (70.9)	39 (37.1)	
Age (years)					<0.0001
24–34		61 (53.5)	50 (63.3)	34 (34)	
35–44		18 (15.7)	17 (21.5)	23 (23)	
45–54		29 (25.4)	8 (10.1)	16 (16)	
55–64		6 (5.3)	4 (5.1)	19 (19)	
Above 65		0	0	8 (8)	
Practice hours					0.363
Full time		97 (82.9)	69 (89.6)	86 (82.7)	
Part time		20 (17.1)	8 (10.4)	18 (17.3)	
Years after graduation					0.004
<5		41 (35.3)	27 (34.2)	28 (26.7)	
5–10		22 (19.0)	24 (30.4)	22 (21.0)	
11–15		11 (9.5)	14 (17.7)	12 (11.4)	
15–20		11 (9.5)	4 (5.1)	7 (6.7)	
20–25		20 (17.2)	5 (6.3)	11 (10.5)	
>25		11 (9.5)	5 (6.3)	25 (23.8)	
Practice type					<0.0001
Private practice		72 (64.3)	44 (59.5)	34 (33.3)	
Public hospital		31 (27.7)	21 (28.4)	68 (66.7)	
Multiple locations		9 (8)	9 (12.2)	0	
Practice region					0.285
Urban		107 (91.5)	71 (89.9)	90 (86.5)	
Suburban		7 (6.0)	7 (8.9)	7 (6.7)	
Rural		3 (2.6)	1 (1.3)	7 (6.7)	
Age of patients					
0–3 years	yes	43 (36.8)	68 (86.1)	86 (84.3)	<0.0001
	no	74 (63.2)	11 (13.9)	16 (15.7)	
3–6 years	yes	69 (59.0)	77 (97.5)	88 (86.3)	<0.0001
	no	48 (41)	2 (2.5)	14 (13.7)	
6–12 years	yes	73 (62.4)	58 (73.4)	85 (83.3)	0.002
	no	44 (37.6)	21 (26.6)	17 (16.7)	
>12 years	yes	101 (86.3)	33 (41.8)	78 (76.5)	<0.0001
	no	16 (13.7)	46 (58.2)	24 (23.5)	
Previous oral health course training					
yes		-	-	57 (54.3)	
no				48 (45.7)	
Source of oral health course (multiple choice)					
University		-	-	29 (27.6)	
Residency				19 (18.1)	
CME				31 (29.5)	

Statistical significance determined through chi-square test (*α* < 0.05); Results may not add up to 100% because of missing data; CME: continuing medical education.

**Table 2:** Distribution of responses to knowledge, attitude, and practice (KAP) questions among three groups.

Knowledge	Responding Correctly			*p* Value *
	General Dentists, *n* (%)	Pediatric Dentists, *n* (%)	Pediatricians, *n* (%)	
Which teeth erupt by 6 years of age?	116 (99.1)	78 (98.7)	24 (22.9) ^a,b^	<0.0001
At what age do children stop teething?	107 (91.5)	78 (98.7)	38 (36.2) ^a,b^	<0.0001
When should the child’s first dental visit occur?	107 (91.5) ^a^	76 (97.4)	78 (74.3) ^a,b^	<0.0001
At what age can children use a smear of fluoride toothpaste?	105 (89.7)	71 (89.9)	53 (50.5) ^a,b^	0.074
At what age can children can start using fluoride mouth rinse?	69 (60.0) ^a^	63 (79.7)	25 (23.8) ^a,b^	<0.0001
Until what age do children not need any fluoride supplement?	90 (77.6) ^a^	73 (92.4)	24 (22.9) ^a,b^	<0.0001
What is the PATF ^†^ with high adherence to teeth and low ingestion possibility?	95 (81.2) ^a^	73 (92.4)	10 (9.5) ^a,b^	<0.0001
What is the dose of PATF?	82 (70.1) ^a^	75 (94.9)	0 (0) ^a,b^	<0.0001
Which of the following statements are incorrect? (*Correct answer: Close meals do not entail a risk of caries*)	116 (99.1)	78 (100.0)	66 (62.9) ^a,b^	<0.0001
Who may take fluoride tablets? (*Correct answer: 7-month-old living in nonfluoridated area*)	50 (42.7) ^a^	55 (71.4)	10 (9.5) ^a,b^	<0.0001
**Practice**	**Responding “Often” or “Very Often” (“Willing” or “Very Willing” for Pediatricians)**			*p* Value *
	**General Dentists, *n* (%)**	**Pediatric Dentists, *n* (%)**	**Pediatricians, *n* (%)**	
Inquire about feeding bottle use	78 (67.2) ^a^	75 (94.9)	79 (76.7) ^a^	<0.0001
Examine child’s teeth for caries	114 (98.3)	79 (100.0)	61 (59.2) ^a,b^	<0.0001
Assess child’s risk	92 (79.3) ^a^	76 (96.2)	61 (58.7) ^a,b^	<0.0001
Assess fluoride intake	40 (34.5) ^a^	60 (75.9)	36 (35.0) ^a^	<0.0001
Provide counseling on tooth brushing	111 (95.7)	78 (98.7)	78 (75.0) ^a,b^	<0.0001
Inquire about parents’ dental health	58 (50.0)	36 (45.6)	68 (65.4) ^a,b^	<0.0001
**Attitude**	**Responding Disagree (vs. Uncertain and Agree)**			*p* Value *
	**General Dentists, *n* (%)**	**Pediatric Dentists, *n* (%)**	**Pediatricians, *n* (%)**	
Activities are not sufficiently important to include in physicians’ daily practice	87 (74.4)	65 (82.3)	78 (75)	0.387
Dentists should perform these activities	13 (11.1)	14 (17.7)	24 (23.1) ^b^	0.060
Learning how to perform these activities is difficult for physicians	50 (42.7)	34 (43.6)	32 (30.8)	0.114
Learning how to perform these activities is time consuming	48 (41)	31 (39.2)	35 (33.7)	0.512
Fluoride varnish is difficult for physicians to apply	53 (45.3)	28 (35.4)	28 (26.9) ^b^	0.018
Physicians have no time for oral screening	35 (29.9)	19 (24.1)	36 (34.6)	0.303
Physicians have no time for caries risk assessment	35 (29.9)	17 (21.5)	34 (32.7)	0.236
Physicians have no time for fluoride varnish application	40 (34.2)	24 (30.4)	17 (16.3) ^a,b^	0.009
Physicians have no time for dentist referral	64 (54.7)	47 (59.5)	79 (76.0) ^a,b^	0.003
Physicians have no time for counseling parents	29 (24.8)	17 (22.4)	52 (50.0) ^a,b^	<0.0001
Physicians are not sufficiently knowledgeable to perform these activities	26 (22.4)	12 (15.4)	29 (27.9) ^a^	0.136
Physicians are not sufficiently confident	20 (17.1)	10 (12.8)	19 (18.3)	0.596
Patients are too young and uncooperative	22 (18.8)	14 (17.7)	52 (50.0) ^a,b^	<0.0001

* Chi-square test analysis (*α* < 0.05) between three groups; ^†^ PATF: professionally applied topical fluoride; ^a^ Statistically significant difference at *p* < 0.05 in chi-square test compared with pediatric dentists; ^b^ Statistically significant difference at *p* < 0.05 in chi-square test compared with general dentists.

**Table 3:** Mean KAP scores in three groups.

	General Dentists	Pediatric Dentists	Pediatricians
K score	8.01 ± 1.42 ^a^	9.11 ± 1.02 ^b^	3.12 ± 1.68
A score	24.17 ± 7.41	24.23 ± 6.73	26.82 ± 6.44 ^c^
P score	23.36 ± 4.16	26.85 ± 2.43 ^b^	22.82 ± 4.96

Statistical significance determined through *t* test; ^a^ Significantly higher score compared with pediatricians (*p* < 0.0001); ^b^ Significantly higher score compared with general dentists and pediatricians (*p* < 0.0001); ^c^ Significantly higher score compared with general and pediatric dentists (*p* < 0.05).

**Table 4:** Distribution of responses to “Do you agree that physicians can apply fluoride varnish to prevent ECC in children?”.

	General Dentists, *n* (%)	Pediatric Dentists, *n* (%)	Pediatricians, *n* (%)	*p* Value *
Agree	40 (34.2)	16 (20.3)	60 (58.3)	<0.0001
Disagree	77 (65.8)	63 (79.7)	43 (41.7)	

* Statistical significance determined through chi-square test.

**Table 5:** Correlation between KAP scores in three groups.

Variables	General Dentists		Pediatric Dentists		Pediatricians	
	r_s_	*p* Value	r_s_	*p* Value	r_s_	*p* Value
Knowledge-Practice	0.271	0.003 **	0.217	0.054	0.262	0.008 **
Knowledge-Attitude	−0.178	0.056	0.052	0.657	0.242	0.013 *
Attitude-Practice	−0.073	0.437	0.072	0.541	0.271	0.006 **

r_s_: Spearman rank correlation coefficient; ** Correlation is significant at <0.01 level; * Correlation is significant at <0.05 level.

**Table 6:** Univariate logistic regression analysis on association of attitude items and disagreement on the statement “Do you agree that physicians can apply fluoride varnish to prevent Early Childhood Care (ECC) in children?”.

Attitude Variable		General Dentists	Pediatric Dentists	Pediatricians
		OR (95% CI)	OR (95% CI)	OR (95% CI)
1. Activities are not sufficiently important to include in pediatric visits	Disagree	ref		
	Uncertain	2.32 (0.46–11.63)	0.42 (0.09–1.92)	1.45 (0.51–4.11)
	Agree	1.35 (0.47–3.88)	0.06 * (0.006-–0.64)	12.7 * (1.48–108.76)
2. Dentists should perform these activities	Disagree	ref		
	Uncertain	0	0.11 (0.01–1.14)	0.77 (0.25–2.4)
	Agree	4.43 * (1.32–14.76)	0.74 (0.14–3.8)	1.37 (0.5–3.77)
3. Learning how to perform these activities is difficult for physicians	Disagree	ref		
	Uncertain	2.0 (0.8–4.97)	2.87 (0.69–11.84)	2.46 (0.88–6.84)
	Agree	7.5 ** (2.3–24.44)	5.02 (0.99–25.34)	2.87 (0.99–8.31)
4. Learning how to perform these activities is time consuming for physicians	Disagree	ref		
	Uncertain	1.73 (0.64–4.66)	2.35 (0.63–8.76)	1.34 (0.5–3.56)
	Agree	4.02 ** (1.55–10.45)	2.45 (0.57–10.43)	1.8 (0.68–4.78)
5. Fluoride varnish is difficult for physicians to apply	Disagree	ref		
	Uncertain	2.33 (0.86–6.29)	3.39 (0.91–12.6)	1.82 (0.66–5.02)
	Agree	6.85 ** (2.31–20.26)	16.1 * (1.9–137.1)	2.67 (0.89–8.02)
6. Physicians have no time for oral screening	Disagree	ref		
	Uncertain	1.57 (0.54–4.54)	-- (Not applicable)	3.08 * (1.09–8.67)
	Agree	2.96 * (1.21–7.26)	3.27 (0.99–10.75)	2.58 (0.95–7.03)
7. Physicians have no time for caries risk assessment	Disagree	ref		
	Uncertain	2.28 (0.72–7.20)	5.33 (0.52–54.34)	2.51 (0.91–6.93)
	Agree	5.12 ** (2.07–12.68)	6.09 ** (1.76–21.04)	2.37 (0.85–6.57)
8. Physicians have no time for fluoride varnish application	Disagree	ref		
	Uncertain	1.90 (0.72–5.02)	3.15 (0.81–12.16)	13.17 * (1.54–112.05)
	Agree	5.38 ** (2.01–14.38)	5.4 * (1.26–23.04)	16.0 ** (1.98–129.29)
9. Physicians have no time for dentist referral	Disagree	ref		
	Uncertain	7.51 * (1.59–35.39)	2.29 (0.45–11.61)	0.42 (0.12–1.44)
	Agree	3.75 ** (1.43–9.83)	1.22 (0.29–5.12)	0.33 (0.06–1.7)
10. Physicians have no time for counseling parents	Disagree	ref		
	Uncertain	1.27 (0.38–4.22)	4.9 (0.83–28.72)	0.88 (0.32–2.39)
	Agree	2.03 (0.82–4.98)	3.6 * (1.02–12.69)	1.26 (0.49–3.27)
11. Physicians are not sufficiently knowledgeable	Disagree	ref		
	Uncertain	2.13 (0.66–6.88)	2.61 (0.47–14.57)	2.48 (0.89–6.91)
	Agree	5.76 ** (2.17–15.27)	3.92 (0.99–15.5)	1.35 (0.48–3.78)
12. Physicians are not sufficiently confident	Disagree	ref		
	Uncertain	3.75 (0.57–24.28)	1.33 (0.2–8.7)	7.08 * (1.4–35.7)
	Agree	3.69 * (1.35–10.07)	3.7 (0.86–15.79)	8.5 ** (1.77–40.71)
13. Patients are too young and uncooperative in medical offices	Disagree	ref		
	Uncertain	1.75 (0.52–5.84)	2.22 (0.41–11.82)	0.94 (0.37–2.39)
	Agree	6.76 ** (2.39–19.1)	2.91 (0.77–11.01)	0.58 (0.2–1.68)

OR: odds ratio; CI: confidence interval; * Statistically significant at *p* < 0.05 level; ** Statistically significant at *p* < 0.01 level.
